# Copy number variations associated with fetal congenital kidney malformations

**DOI:** 10.1186/s13039-020-00481-7

**Published:** 2020-03-24

**Authors:** Meiying Cai, Na Lin, Linjuan Su, Xiaoqing Wu, Xiaorui Xie, Ying Li, Xuemei Chen, Yuan Lin, Hailong Huang, Liangpu Xu

**Affiliations:** Department of the Prenatal Diagnosis Center, Fujian Provincial Maternity and Children’s Hospital, affiliated hospital of Fujian Medical University, Fujian Key Laboratory for Prenatal Diagnosis and Birth Defect, Fuzhou, China

**Keywords:** Copy-number variations, Etiology, Chromosomal microarray analysis, Renal hypodysplasia

## Abstract

**Background:**

Congenital anomalies of the kidney and urinary tract (CAKUT) constitute 20–30% of all congenital malformations. Within the CAKUT phenotypic spectrum, renal hypodysplasia (RHD) is particularly severe. This study aimed to evaluate the applicability of single-nucleotide polymorphism (SNP) array test in prenatal diagnosis of RHD for improving prenatal genetic counseling and to search for evidence of a possible causative role of copy-number variations (CNVs) in RHD.

**Results:**

We performed a systematic survey of CNV burden in 120 fetuses with RHD: 103 cases were isolated RHD and 17 were non-isolated RHD. Single-nucleotide polymorphism (SNP) array test was performed using the Affymetrix CytoScan HD platform. All annotated CNVs were validated by fluorescence in situ hybridization. We identified abnormal CNVs in 15 (12.5%) cases of RHD; of these CNVs, 11 were pathogenic and 4 were variants of uncertain significance. The detection rate of abnormal CNVs in non-isolated RHD was higher (29.4%, 5/17) than that in isolated RHD (9.7%, 10/103) (*P* = 0.060). Parents are more inclined to terminate the pregnancy if the fetuses have pathogenic results of the SNP-array test.

**Conclusions:**

The variable phenotypes that abnormal CNVs may cause indicate the genetic counseling is needed for RHD cases.

## Background

Congenital anomalies of the kidney and urinary tract (CAKUT) constitute 20–30% of all congenital malformations, and their prevalence is between three and seven per 1000 births [1]. These malformations account for 40–50% of children’s kidney diseases and 7% of adult end-stage kidney diseases worldwide [2]. Within the CAKUT phenotypic spectrum, renal aplasia, agenesis, hypoplasia, and dysplasia (referred as renal hypodysplasia [RHD]) [3] are particularly severe conditions, affecting 0.5% of the general population [4]. The etiology of the majority of RHD cases remains unknown. However, many studies suggest that the pathogenesis of these birth defects is related to strong genetic factors.

Exploring the potential genetic etiology of congenital anomalies can establish a genetic diagnosis and/or improve the management of possible complications. A few postnatal cases with congenital RHD have been reported to be associated with CNVs [3], which drew our attention to the underlying utility of SNP-array test in the prenatal diagnosis of congenital RHD; we came up with the idea that it could be a genetic disease rather than a sporadic one. Thus, the prognosis of congenital RHD may not be as good as initially believed. In this study, we performed a systematic investigation of abnormal CNVs in fetuses with congenital RHD using Chromosomal Microarray Analysis (CMA).

## Methods

### Patient data

We conducted a retrospective study on RHD cases diagnosed prenatally by fetal ultrasound from the Prenatal Diagnosis Center of Fujian Provincial Maternal and Children Health Hospital, between January 2016 and July 2019. The inclusion criteria included the presence of a renal parenchymal defect such as renal agenesis or renal dysplasia (discovery of a small or cystic kidney for gestational age kidneys) (seven cases have been previously included in our papers such as case 1, case 2, case 8, case 9, case 12, case 13, and case 15) [5].

A total of 120 RHD cases were included: 103 cases of isolated RHD and 17 cases of non-isolated RHD. All included cases underwent invasive prenatal genetic testing, and we preserved qualified DNA samples. Isolated RHD was defined as a renal parenchymal defect such as renal agenesis, hypoplasia, or dysplasia. Non-isolated RHD was defined as RHD with the presence of significant extra-renal malformations. Extra-renal malformations detected in non-isolated RHD cases included cardiac defects (e.g., ventricular septal defect, aortic stenosis, pulmonary valve stenosis, persistent left superior vena cava, tricuspid regurgitation), urorectal septum malformation sequence (URSMS), fetal growth restriction (FGR), nasal bone dysplasia, and single umbilical artery. Approximately 2.0 mL of peripheral venous blood in ethylenediaminetetraacetic acid (EDTA) was collected from the parents if the RHD fetal CNVs detected were considered rare. DNA was extracted using a Gentra Puregene Blood Kit (Qiagen, Hilden, Germany).

Amniocentesis (in 74 cases) and umbilical cord blood collection (in 46 cases) were performed, according to gestational age. Amniotic fluid was collected by amniocentesis at 16–24 weeks of gestation, and fetal blood was collected by cordocentesis after 24 weeks of gestation [5]. The fetuses underwent routine ultrasonic scans, and fetal biometry was assessed at a median gestational age of 24 ± 6 weeks (range: 18 ± 2 to 34 ± 1 weeks). Clinical follow-up was assessed by physical examination and postpartum ultrasound.

### Single-nucleotide polymorphism array

Single-nucleotide polymorphism (SNP) array technology was performed as previously reported by our laboratory [6]. The CNVs were classified into three categories: pathogenic, variants of uncertain clinical significance (VUS), and benign, according to the American College of Medical Genetics guidelines [7]. Parental testing was performed for fetuses with RHD that had abnormal SNP-array results to determine the inheritance pattern of the deletions and/or duplications. All annotated CNVs were experimentally validated by fluorescence in situ hybridization.

### Statistical analysis

Statistical analysis was performed with SPSS version 20 (IBM, Armonk, NY, USA). Comparisons between isolated RHD and RHD associated with other anomalies were performed using the Fisher’s exact test. A *P* value < 0.05 was considered statistically significant.

## Results

A total of 120 RHD cases were included, of which 103 (85.8%) cases were isolated RHD and 17 (14.2%) were non-isolated RHD (Table 1). We identified abnormal CNVs in 15 (12.5%) cases of RHD; of these 15 CNVs, 11 were pathogenic and 4 were VUS. We also detected two benign CNVs. The detection rate of abnormal CNVs in the non-isolated RHD cases was higher (29.4%, 5/17) than that in the isolated RHD cases (9.7%, 10/103) (*P* = 0.060).

**Table 1:** Phenotypic characteristics of 120 renal hypodysplasia fetuses

Classification	Number of fetuses	Number of CMA		
		Pathogenic CNVs	VUS	Benign
Isolated RHD	103	7	3	2
Non-isolated RHD	17	4	1	0
Total	120	11	4	2

*VUS* Variation of uncertain clinical significance

### Abnormal CNVs in fetuses with isolated RHD

Of the 103 cases of isolated RHD, 7 were pathogenic and 3 were VUS. The CMA nomenclature, pathogenic renal genes, and inheritance status are described in Table 2. Seven pathogenic CNVs (one 22q11.2 deletion, four 17q12 deletion, one 17p12 deletion, and one 3q28 deletion) were identified in seven fetuses with isolated RHD. We detected a de novo 22q11.21 deletion in case 1, which had an ectopic right kidney with dysplasia deformations.

**Table 2:** Ten abnormal copy-number variations detected in fetuses with isolated renal hypodysplasia

case	CMA results	Size (Mb)	Renal phenotype	Pathogenicity classification	Obstetrical outcomes	Inheritance
1	arr [hg19]22q11.21 (18,916,842-21,800,471) × 1	2.8	ectopic right kidney with dysplasia	p	TP	de novo
2	arr[hg19]17q12(34,822,465-36,311,009) × 1	1.4	left renal dysplasia	p	TP	de novo
3	arr[hg19]17q12(34,822,465-36,243,365) × 1	1.4	kidney echo enhancement	p	TP	de novo
4	arr[hg19]17q12(34,822,465-36,307,773) × 1	1.48	kidney echo enhancement	p	TP	de novo
5	arr[hg19]17q12(34,822,465-36,404,555) × 1	1.58	kidney echo enhancement	p	TP	de novo
6	arr[hg19]17p12(14,083,054-15,482,833) × 1	1.4	left renal agenesis	p	TP	Maternal
7	arr[hg19]3q28(188,788,120-191,331,505) × 1,15q11.2 (23,620,191-24,978,547) × 3	2.5 1.3	right renal agenesis	p	TP	de novo
8	arr[hg19]9q21.31q21.32 (82,732,469-85,502,241) × 1	2.7	left renal dysplasia	VUS	TD	de novo
9	arr[hg19]2q31q34(111,859,545-209,533,369) × 2–3	97.9	renal dysplasia	VUS	TD	de novo
10	arr[hg19]3p26.1p24.1 (8,494,626-26,413,121)hmz	17.9	right renal agenesis	VUS	TD	Not reported

*P* Pathogenic, *TD* Term delivery, *TP* Termination of pregnancy, *VUS* Variation of uncertain clinical significance

Three VUS CNVs were identified in fetuses with isolated RHD. We found case 8 with a 9q21.31q21.32 deletion in a region including the *TLE1* gene [8]. At 20 weeks of gestation, this case presented with left renal dysplasia, and the finding was regarded as VUS. At 22 weeks, case 9 presented with renal dysplasia, and its SNP-array test showed a partial duplication of 2q31q34. At 27 weeks of gestation, we identified case 10 with isodisomy, but the ultrasonic diagnosis was presented with right renal dysplasia.

### Abnormal CNVs in fetuses with non-isolated RHD

In the fetuses with non-isolated RHD, the SNP-array test identified clinically significant pathogenic CNVs, involving the isodisomy in 16q23.2q24.3 and 16p13.3p12.3, 22q11.2 deletion, 4p16.3p15.1 deletion, and 7q11.23 duplication (Table 3).

**Table 3:** Five abnormal copy-number variations detected in fetuses with non-isolated renal hypodysplasia

case	CMA results	Size (Mb)	Prenatal ultrasound	Pathogenicity classification	Obstetrical outcomes	Inheritance
11	arr[hg19]22q11.21 (20,730,143-21,800,471) × 1	1.0	left renal dysplasia; Left choroid plexus cyst; strephenopodia	*P*	TP	de novo
12	arr[hg19]16q23.2q24.3 (79,800,878-90,146,366)hmz,16p13.3p12.3 (94,807-19,302,326)hmz	10.3	left renal agenesis; VSD; PVS; FGR	*p*	TP	UPD
13	arr[hg19]4p16.3p15.1 (68,345-35,252,743) × 1	35	renal hypoplasia;FGR; nasal bone dysplasia	*P*	TP	de novo
14	arr[hg19]7q11.23 (72,701,098-74,069,645) × 3	1.3	left renal agenesis, VSD;	*P*	TP	de novo
15	arr[hg19]16p13.11 (15,325,072-16,272,403) × 3	0.92	left renal dysplasia;URSMS	VUS	TP	de novo

*FGR* Fetal growth restriction, *p* Pathogenic, *PVS* Pulmonary valve stenosis, *TP* Termination of pregnancy, *UPD* Uniparental disomy, *URSMS* Urorectal septum malformation sequence, *VSD* Ventricular septal defect, *VUS* Variation of uncertain clinical significance

### Inheritance analysis

We screened the inheritance information of 14 families with abnormal CNVs (the parents of one case refused SNP-array test). The analysis showed that two fetuses inherited abnormal CNVs from unaffected parents, whereas 12 were de novo CNVs.

### Obstetrical outcomes and clinical follow-up

We received delivery information from 120 pregnancies with RHD. Parental analysis showed that the abnormalities occurred de novo in 12 fetuses, CNV was inherited from unaffected parents in two cases, and the parents of one case refused SNP-array test. The reasons for the termination of pregnancies in the 11 women analyzed were pathogenic CNVs. We also found that one VUS CNV case (due to fetal URSMS) and two normal CNVs cases of RHD with extra-renal defects (due to severe fetal malformations) resulted in the termination of the pregnancy. Clinical follow-up assessments and postpartum ultrasound were performed at 42 days after birth.

## Discussion

Fetal malformations represent a poorly studied group of developmental disorders. The significant etiological heterogeneity of congenital kidney malformations cannot be detected by clinical evaluation, and because most structural variations are below the resolution of cytogenetic analysis, high-resolution genomic methods are required. Our objective was to assess the impact of CMA on the investigation of fetal congenital kidney malformations.

Many studies suggest that CNVs contribute to the etiology of RHD and implicate the genes within the CNV loci (e.g., *PAX2*, *HNF1B, KIF26B*, which are associated with kidney defects), that are detected in up to 10% of individuals with kidney malformations [9–12]. We detected 15 distinct known or novel CNVs in 12.5% of RHD cases, indicating a large proportion of rare pathogenic CNVs in fetal congenital kidney malformations. The overall diagnostic yield of 12.5% is lower than that obtained with the use of CMA for the investigation of rare disorders in children or adults, which is 17.2% [3]. The lower rate of CMA for fetal congenital kidney malformations may be related to the involvement of epigenetic mechanisms or more complex genetic models.

Seven cases of pathogenic CNVs were related to kidney development in our study. The most frequent genomic imbalances that we detected in isolated RHD cases were 22q11.2 and 17q12 deletion. According to previous studies, congenital kidney, and urinary tract anomalies are present in approximately 30% of the patients with 22q11.21 deletion [13]. In this study, we found two cases with 22q11.21 deletion. Four cases had 17q12 deletion, their genomic regions involved hepatocyte nuclear factor 1 homeobox B (*HNF1B*), associated with renal cyst and diabetes (RCAD) syndrome [14]. We found two de novo pathogenic CNVs, a duplication in 15q11.2 and a deletion in 3q28, and identified two genes (*TP63* and *CLDN16)*. Per the literature [15], *TP63* and *CLDN16* genes are related to kidney development and developmental delay.

Four cases of pathogenic CNVs were likely related to kidney development. A deletion in 17p12 results in hereditary neuropathy with liability to pressure palsy [16]. According to the previous studies, there are no reports indicating that 17p12 deletion syndrome is related to RHD. Based on the DECIPHER database (http://www.sanger.ac.uk/PostGenomics/decipher/), DGV database (http://projects.tcag.ca/variation), and OMIM database (http://www.omim.org), 17p12 deletion was reported as clinically significant pathogenic CNVs. However, kidney-related genes were not found in chromosome 17p12. In one uniparental disomy case, the isodisomy in 16q23.2q24.3 and 16p13.3p12.3 was detected, of size 19.2 Mb, and containing 179 OMIM genes. CMA on the parents revealed maternal uniparental disomy (UPD). Due to the used test we cannot exclude heterodisomy. Both heterodisomy and isodisomy can cause a disease if they affect a gene underlying genomic imprinting. In addition, isodisomy can result in functional loss to heterozygosity. Isodisomy which is inheritance of two copies of one parental chromosome, has same type of SNP sites results in the loss of heterozygosity can be detected according to the allele different lines. Heterodisomy inheritance of two homologous but genetically different chromosomes from one parent, unable to distinguish with only result of fetal. It can only be detected by pedigree linkage analysis. According to the UPD database (Liehr T. 2020. Cases with uniparental disomy. http://cs-tl.de/DB/CA/UPD/0-Start.html [accessed 01/01/2020]) and literatures [17], the UPD on Chr16 was identified with pathogenic CNVs and the phenotypes mainly included intrauterine growth retardation, cardiac malformation, and urinary system malformation. However, kidney-related genes were not identified in the UPD on Chr16. A de novo pathogenic deletion in 4p of 35 Mb and containing 105 OMIM genes was identified. This region includes the key gene for Wolf-Hirschhorn syndrome (WHS). WHS is a well-known syndrome related to subtelomeric deletions in the short arm of chromosome 4 [18]. We also identified a 1.3 Mb de novo duplication, resulting in 7q11.23 duplication syndrome. 7q11.23 duplication syndrome is caused by the reciprocal duplication of the 26 genes that are deleted in Williams syndrome. In the past few years, many cases with the classic 7q11.23 duplication syndrome have been reported in the literature [19–22]. These case reports have provided a detailed phenotypic description of 7q11.23 duplication syndrome and highlighted its variability. Whether these four cases of pathogenic CNVs were related to RHD still needs more clinical data and biological function verification in the future.

Four VUS were identified that were likely related to kidney development. In the deletion of chromosome 9, *TLE1* appears to be a gene of interest; it has also been reported as a risk factor for synovial sarcoma [23]. Further investigation is needed to determine whether this deletion has an effect on kidney development. The second VUS is a mosaicism of q13q34 region in chromosome 2 with a rate of about 58%, and a duplication of the 97.9 Mb fragment. Some reports have suggested the possibility that the overexpression of a gene(s) on q13q34 region in chromosome 2 may cause developmental delay and orofacial clefting [24]. The mosaicism of q13q34 region in chromosome 2 has not been reported in the current literature, and our SNP-array test show that the parents of proband are normal. Therefore, the clinical significance of this CNV is not yet clear. The third VUS is a de novo duplication in 16p13.11. Although this variant does not explain the left renal dysplasia, it could be a risk factor for neurodevelopmental disorders and colorectal tumorigenesis [25, 26]. However, there are rare reports of 16p13.11 duplication casing a renal defect [27, 28]. Whether or not 16p13.11 duplication is associated with renal development still needs to be further verified with larger samples. The fourth VUS is isodisomy (due to the used test we cannot exclude heterodisomy) in 3p26.1p24.1, of size 17.9 Mb size and containing 83 OMIM genes. The isodisomy increases the risk of recessive genetic disease. The parents refused SNP-array test in the genes, so we defined the clinical implications of isodisomy in 3p26.1p24.1 as VUS variation.

We observed that eleven pregnant women with fetuses with pathogenic CNVs chose to terminate the pregnancy. One VUS CNV case (due to severe fetal URSMS) and two normal CNVs cases of RHD with extra-renal defects (due to severe fetal malformations) still terminated their respective pregnancies. The identification of pathogenic CNVs can improve genetic counseling, and raise the awareness of parents about RHD to reduce unnecessary terminations of pregnancy.

Owing to the retrospective nature of our study, it has some limitations. Determining the link between a CNV and the observed phenotype is complicated. Perhaps the application of some new genetic techniques could indicate the presence of other genetic anomalies that remained undetected. In addition, an important component of prenatal consultation for the patients is counseling on the long-term physical and mental development associated with RHD, which cannot be obtained from our study.

## Conclusion

The clinical application of CMA plays an important role in the judgment of fetal prognosis. Parents are more inclined to terminate the pregnancy if the fetuses have pathogenic results of the SNP-array test. After CMA excluded the genetic diseases related to pathogenic CNVs in the fetus, fetal MRI is feasible to evaluate urinary systems such as fetal kidney, ureter, and bladder. Meanwhile, multidisciplinary cooperation is conducted with pediatric surgery to formulate a diagnosis and treatment plan for the fetus after birth.

## Data Availability

The datasets used and/or analyzed during the current study are available from the corresponding author on reasonable request.

## Abbreviations

CAKUT: Congenital malformations of the kidney and urinary tract
CNVs: Copy number variations
CMA: Chromosomal microarray analysis
FGR: Fetal growth restriction
LOH: Loss of heterozygosity
FISH: Fluorescence in situ hybridization
PCR: Polymerase chain reaction
RHD: Renal hypodysplasia
SNP: Single nucleotide polymorphism
URSMS: Urorectal septum malformation sequence
UPD: Uniparental disomy
VUS: Variation of uncertain clinical significance
